# Disease-specific health-related quality of life (HRQL) instruments for food allergy: protocol for a systematic review

**DOI:** 10.1186/2045-7022-3-15

**Published:** 2013-05-01

**Authors:** Sarah A Salvilla, Sukhmeet S Panesar, Shyamal Patel, Tamara Rader, Antonella Muraro, Graham Roberts, Bertine Flokstra de-Blok, Anthony Dubois, Aziz Sheikh

**Affiliations:** 1 University of Edinburgh, Teviot Place, Edinburgh EH8 9AG, UK; 2 St. George’s University, Cranmer Terrace, London SW17 0RE, UK; 3 University of Ottawa, 75 Laurier Avenue East, Ottawa, ON K1N 6N5, Canada; 4 Padua General University Hospital, Via Giustiniani 3, Padua 35128, Italy; 5 Faculty of Medicine, University of Southampton, Southampton SO171BJ, UK; 6 Department of General Practice, GRIAC Research Institute, University Medical Center Groningen, PO Box 30.001, Groningen 9700 RB, The Netherlands; 7 Department of Paediatrics, University Medical Centre Groningen, Groningen 9700 RB, The Netherland; 8 Division of Paediatric Pulmonology and Paediatric Allergy, University Medical Centre Groningen, PO Box 30.001, Groningen 9700 RB, The Netherland; 9 University of Groningen, Groningen 9700 RB, The Netherland; 10 Centre for Population Health Sciences, The University of Edinburgh Medical School, Doorway 3, Teviot Place, Edinburgh EH8 9AG, UK

**Keywords:** Food allergy, IgE-mediated, QOL, Quality of life

## Abstract

**Background:**

The European Academy of Allergy and Clinical Immunology is in the process of developing its *Guideline for Food Allergy and Anaphylaxis*, and this systematic review is one of seven inter-linked evidence syntheses that are being undertaken in order to provide a state-of-the-art synopsis of the current evidence base in relation to epidemiology, prevention, diagnosis and clinical management, and impact on quality of life, which will be used to inform clinical recommendations. The aim of this systematic review will be to determine which validated instruments can be employed to enable assessment of the impact of, and investigations and interventions for, food allergy on health-related quality of life.

**Methods:**

Seven bibliographic databases were searched from their inception to September 30, 2012 for disease-specific HRQL questionnaires that were specifically designed for use with patients/carers and any articles relating to the description, development and/or the validation of the above identified HRQLs. There were no language or geographic restrictions. We will assess the development of the instruments identified and their performance properties including: validity; generalizability; responsiveness; managing missing data; how variation in patient demography was managed; and cross-cultural and linguistic adaptation, using a previously reported quality assessment tool.

**Discussion:**

Using appropriately developed and validated instruments is critical to the accurate evaluation of HRQL in people with food allergy. This review will systematically appraise the evidence on the subject and help to identify any gaps.

## Background

The umbrella term ‘food hypersensitivity’ can be used to describe any ‘adverse reaction to food’ [1]. The term ‘food allergy’ refers to the subgroup of food-triggered reactions in which immunologic mechanisms have been implicated, whether IgE-mediated, non-IgE-mediated, or involving a combination of IgE- and non-IgE-mediated etiologies [2]. All other reactions to food that were in the past sometimes referred to as ‘food intolerance’ constitute non-allergic food hypersensitivity reactions and are outside the focus of this systematic review. Coeliac disease is an important non-IgE mediated condition but as it has distinct symptoms and prognosis different from atopic conditions it will be excluded from this review [3].

IgE-mediated reactions can, for example, manifest as angioedema, urticaria, atopic eczema/dermatitis, oral allergy syndrome and anaphylaxis. Non-IgE-mediated immunologic reactions result from activation of other immunologic pathways (e.g. T-cell mediated) and can manifest as atopic eczema/dermatitis, gastro-esophageal reflux disease, food protein-induced enterocolitis, proctocolitis, and enteropathy syndromes. The contemporary definition of food allergy thus includes several clinical entities with different pathophysiologies [3], resulting from exposure to different foods. This review will consider only instruments developed for IgE-mediated food allergy.

There are a number of concerns regarding outcome measures in food allergy: the vast array of outcome measures used in research settings; their specificity, i.e. related to one condition rather than the whole person; gaps in relation to allergic conditions other than asthma, eczema and rhinitis; and the fact that they do not always include issues that are pertinent to patients [4]. Living with a food allergy is more difficult than is generally appreciated. Management is focused on the avoidance of food causing allergic reactions, which places a psychological burden on patients that is associated with high stress levels and anxiety. In some cases, this can have a considerable impact on their daily lives. Quality of life as defined by the World Health Organization (WHO) is “the individual's perception of their position in life in the context of the culture and value systems in which they live and in relation to their goals, expectations, standards and concerns” [5]. The importance of measuring quality of life in patients is that such measurement allows for the estimation of the impact of the disease from a patient perspective, as it is possible that two individuals with clinically similar disease severity may assess impairment in their everyday lives to different degrees [6]. Therefore quality of life measurements are being utilised by clinicians to gain insights into specific problems patients face and the effectiveness of interventions to enhance patient’s quality of life [7].

Health-related quality of life can be measured using generic or disease specific questionnaires. Useful attributes of generic quality of life questionnaires are that they allow comparison between different diseases as well as being sensitive to co-morbidities. However, associated limitations of generic instruments include the fact that they are less sensitive than disease-specific questionnaires, hence potentially important differences or changes may be missed. This is particularly seen in the context of food allergy, where unless individuals are exposed to the specific food, patients will have no symptoms or problems other than the need to avoid certain foods [8]. The disease-specific questionnaires that have been developed are significantly more sensitive in measuring the response to interventions or future treatments, as well as estimating the general burden of food allergy [9].

The European Academy of Allergy and Clinical Immunology (EAACI) is in the process of developing the *EAACI Guideline for Food Allergy and Anaphylaxis*, and this systematic review is one of seven inter-linked evidence syntheses that are being undertaken in order to provide a state-of-the-art synopsis of the current evidence base in relation to epidemiology, prevention, diagnosis and clinical management, and impact on quality of life, which will be used to inform clinical recommendations.

### Aims

The aims of this systematic review will be to determine which validated instruments can be employed to enable assessment of the impact of, and investigations and interventions for, food allergy on health-related quality of life.

## Methods

### Search strategy

**Figure 1:**
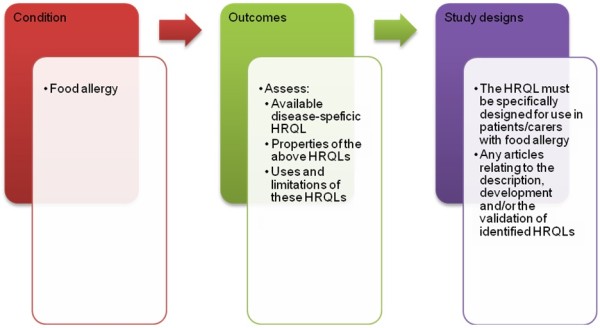
Conceptualisation of systematic review of disease-specific health-related quality of life (HRQL) tools for food allergy.

A sensitive search strategy will be designed to retrieve all articles combining the concepts of food allergy and quality of life, and patient reported outcomes from electronic bibliographic databases. We have conceptualised the search to incorporate three elements, as shown in Figure 1: Conceptualisation of systematic review of disease-specific health-related quality of life (HRQL) tools for food allergy.

We have adapted a search filter from York University Centre for Reviews and Dissemination to retrieve quality of life and patient-reported outcome material. We will search the following databases:

•MEDLINE (OVID)

•Embase (OVID)

•CINAHL (Ebscohost)

•ISI Web of Science (Thomson Web of Knowledge)

The search strategy has been devised on OVID MEDLINE and then adapted for the other databases (see Additional file 1 for full search strategies). In all cases, the databases will be searched from 1990 to 30 September 2012. All references will be imported into an EndNote Library and tagged with the name of the database. Searches will be limited to literature from 1990 onwards; this limit is based on the first publication of key allergy HRQLs such as the Rhinoconjunctivitis Quality of Life Questionnaire and Asthma Quality of Life Questionnaire [10,11]. Additional references will be located through searching the references cited by the identified studies, and unpublished work and research in progress will be identified through discussion with experts in the field. We will invite experts who are active in the field from a range of disciplines and locations to comment on our search strategy and the list of included studies. There are no language restrictions and, where possible, all literature will be translated.

### Inclusion criteria

•The disease-specific HRQL questionnaire must be specifically designed for use in patients/carers with food allergy

•Any articles relating to the description, development and/or the validation of the above identified HRQLs.

### Exclusion criteria

•Reviews, discussion papers, non-research letters and editorials

•Case studies and case series

•Animal studies.

### Study selection

The titles will be checked independently by two reviewers according to the above selection criteria and categorised as: included, not included and unsure. For those papers in the unsure category, we will retrieve the abstract and re-categorise as above. Any discrepancies will be resolved by consensus and, if necessary, a third reviewer will be consulted to arbitrate. Full text copies of potentially relevant studies will be obtained and their eligibility for inclusion independently assessed. Studies that do not fulfil all of the inclusion criteria will be excluded.

### Quality assessment strategy

We plan to assess the development of the instruments identified and their performance properties, including validity, generalisability, responsiveness, managing missing data, how variation in patient demography is managed, cross-cultural and linguistic adaptation using a previously reported quality assessment tool [12]. Assessment of validity will focus on identification of appropriate independent measures and their correlation with partial or total instrument scores. A team of researchers will independently assess the articles against the defined criteria and any discrepancies will be resolved by consensus and, if necessary, a third reviewer will be consulted.

### Analysis, data synthesis and reporting

Data will be independently extracted onto a customised data extraction sheet by two reviewers, and any discrepancies will be resolved by discussion or, if agreement cannot be reached, by arbitration by a third reviewer.

A descriptive summary with data tables will be produced to summarise the literature. If clinically and statistically appropriate, meta-analysis using either fixed-effect or random-effects modelling will be undertaken using methods suggested by Agresti and Coull [13]. A narrative synthesis of the data will also be undertaken.

This review has been registered with the International Prospective Register of Systematic Reviews (PROSPERO) and has registration number CRD42013003710 allocated to it. The Preferred Reporting Items for Systematic Reviews and Meta-Analyses (PRISMA) checklist will be used to guide the reporting of the systematic review [14].

## Discussion

Food allergy has a profound impact on children, adolescents and their families. In particular, the constant vigilance needed to avoid allergens and the daily management of food allergy impacts on daily family activities and social events. The main strengths of this work include the development of a detailed systematic review protocol, a comprehensive search strategy and a detailed critical appraisal of the identified instruments. The limitations of this work include the fact that our focus will be on IgE-mediated food allergy, whereas we know that non-IgE-mediated and mixed IgE/non-IgE-mediated food allergy is common (particularly in young children) and can have a profound impact on QOL. This decision reflects the expert opinion that such instruments have yet to be developed; this therefore reflects an important outstanding research need.

## Abbreviations

EAACI: European Academy of Allergy and Clinical Immunology; HRQL: Health-related quality of life; PROSPERO: Prospective Register of Systematic Reviews; PRISMA: Preferred Reporting Items for Systematic Reviews and Meta-Analyses; WHO: World Health Organization

## Competing interests

The authors declare that they have no competing interests.

## Authors’ contributions

SS, SSP, SP and TR conceptualised and designed the protocol and drafted earlier versions of the document in their capacity as methodologists. AM, GR, BFdeB and AD contributed to further refinements of the protocol and revised it critically for important intellectual content in their capacity as guideline leads. AS led on the development of concepts used in this protocol and revised it critically for important intellectual content in his capacity as the methodology lead. All authors approved the final version to be published.

## Supplementary Material

Additional file 1 Search strategies. Click here for file
