# Supplementary material for: Disease-specific health-related quality of life (HRQL) instruments for food allergy: protocol for a systematic review
Source: Clin Transl Allergy. 2013 May 1;3:15. doi: 10.1186/2045-7022-3-15 (PMC3651316; doi:10.1186/2045-7022-3-15)
Supplement: Additional file 1 — Search strategies. [file 2045-7022-3-15-S1.docx]

**Additional file 1: Search strategies**

*Database: Ovid MEDLINE(R) In-Process & Other Non-Indexed Citations and Ovid MEDLINE(R) <1946 to Present>*

Search Strategy:

--------------------------------------------------------------------------------

| 1 | exp Food Hypersensitivity/ |
| --- | --- |
| 2 | foodallerg*.mp. |
| 3 | food hypersensitivity.mp. |
| 4 | food hypersensitivities.mp. |
| 5 | allergy, food.mp. |
| 6 | or/1-5 |
| 7 | (rat or rats or cow or cows or chicken? or horse or horses or mice or mouse or bovine or animal?).ti. |
| 8 | (animal$ not human$).sh,hw. |
| 9 | 7 or 8 |
| 10 | 6 not 9 |
| 11 | Quality of Life/ |
| 12 | QOL.ti,ab. |
| 13 | Health Status/ |
| 14 | Health Status Indicators/ |
| 15 | Activities of Daily Living/ |
| 16 | Quality-Adjusted Life Years/ |
| 17 | (quality adj4 life).ti,ab. |
| 18 | (well adj4 being).ti,ab. |
| 19 | HRQL.tw. |
| 20 | QALY$.tw. |
| 21 | (Health$ adj2 state).ti,ab. |
| 22 | (Life adj3 quality).ti,ab. |
| 23 | (Health$ adj2 year$ adj2 equivalent$).ti,ab. |
| 24 | (subjective adj2 health adj2 status).ti,ab. |
| 25 | (Health Care)"/ |
| 26 | (patient adj2 reported adj2 outcome adj2 measure$).ti,ab. (530) |
| 27 | (patient adj2 outcome).ti,ab. |
| 28 | patient preference$.ti,ab. |
| 29 | *Patient Participation/ |
| 30 | *"Patient Acceptance of Health Care"/ |
| 31 | consumersatisfaction.ti,ab. |
| 32 | *Patient Satisfaction/ |
| 33 | *Questionnaires/ |
| 34 | *Health Surveys/ |
| 35 | tool$.ti,ab. |
| 36 | *Psychometrics/ |
| 37 | Utilit$.ti,ab. |
| 38 | Short form 36.mp. |
| 39 | Short form 12.mp. |
| 40 | (SF 12 or SF 36 or SF-12 or SF-36).ti,ab. |
| 41 | Euroqol.mp. |
| 42 | EQ-5D.tw. |
| 43 | Health Utilities Index.mp. |
| 44 | HUI.mp. |
| 45 | Medical Outcomes Survey.mp. |
| 46 | MOS.tw. |
| 47 | QWB.tw. |
| 48 | Rosser.mp. |
| 49 | *"Reproducibility of Results"/ |
| 50 | valid$.ti,ab. |
| 51 | reliab$.tw. |
| 52 | (effect adj1 size).ti,ab. |
| 53 | (Sensitiv$ adj2 change).tw. |
| 54 | Reproduc$.ti,ab. |
| 55 | Utility measure$.ti,ab. |
| 56 | or/11-55 |
| 57 | 10 and 56 |

*Database: Embase <1980 to 2012 Week 32>*

Search Strategy:

| 1 | exp Food Hypersensitivity/ |
| --- | --- |
| 2 | foodallerg*.mp. |
| 3 | food hypersensitivity.mp. |
| 4 | food hypersensitivities.mp. |
| 5 | allergy, food.mp. |
| 6 | or/1-5 (21168) |
| 7 | (rat or rats or cow or cows or chicken? or horse or horses or mice or mouse or bovine or animal?).ti. (1586460) |
| 8 | (animal$ not human$).sh,hw. |
| 9 | 7 or 8 |
| 10 | 6 not 9 |
| 11 | Quality of Life/ |
| 12 | QOL.ti,ab. |
| 13 | Health Status/ |
| 14 | Health Status Indicators/ |
| 15 | Activities of Daily Living/ |
| 16 | Quality-Adjusted Life Years/ |
| 17 | (quality adj4 life).ti,ab. |
| 18 | (well adj4 being).ti,ab. |
| 19 | HRQL.tw. |
| 20 | QALY$.tw. |
| 21 | (Health$ adj2 state).ti,ab. |
| 22 | (Life adj3 quality).ti,ab. |
| 23 | (Health$ adj2 year$ adj2 equivalent$).ti,ab. |
| 24 | (subjective adj2 health adj2 status).ti,ab. |
| 25 | *"Outcome Assessment (Health Care)"/ |
| 26 | (patient adj2 reported adj2 outcome adj2 measure$).ti,ab. |
| 27 | (patient adj2 outcome).ti,ab. |
| 28 | patient preference$.ti,ab. |
| 29 | *Patient Participation/ |
| 30 | *"Patient Acceptance of Health Care"/ |
| 31 | consumersatisfaction.ti,ab. |
| 32 | *Patient Satisfaction/ |
| 33 | or/11-32 |
| 34 | *Questionnaires/ |
| 35 | *Health Surveys/ |
| 36 | tool$.ti,ab. |
| 37 | *Psychometrics/ |
| 38 | Utilit$.ti,ab. |
| 39 | Short form 36.mp. |
| 40 | Short form 12.mp. |
| 41 | (SF 12 or SF 36 or SF-12 or SF-36).ti,ab. |
| 42 | Euroqol.mp. |
| 43 | EQ-5D.tw. |
| 44 | Health Utilities Index.mp. |
| 45 | HUI.mp. |
| 46 | Medical Outcomes Survey.mp. |
| 47 | MOS.tw. |
| 48 | QWB.tw. |
| 49 | Rosser.mp. |
| 50 | *"Reproducibility of Results"/ |
| 51 | valid$.ti,ab. |
| 52 | reliab$.tw. |
| 53 | (effect adj1 size).ti,ab. |
| 54 | (Sensitiv$ adj2 change).tw. |
| 55 | Reproduc$.ti,ab. |
| 56 | Utility measure$.ti,ab. |
| 57 | or/34-56 |
| 58 | 33 and 57 |
| 59 | 10 and 58 |
| 60 | limit 59 to yr="1990 - 2012" |

*Database: CINAHL – Ebscohost*Search strategy:

| S24 | S10 and S23 |
| --- | --- |
| S23 | S11 or S12 or S13 or S14 or S15 or S16 or S17 or S18 or S19 or S20 |
| S20 | (MH "Short Form-36 Health Survey (SF-36)") OR "(SF 12 or SF 36 or SF-12 or SF-36)" |
| S19 | "EQ-5D" |
| S18 | "Euroqol" |
| S17 | "patient preferences" |
| S16 | (MH "Health Status Indicators") OR (MH "Health Status") |
| S15 | (MH "Quality-Adjusted Life Years") |
| S14 | "patient reported outcomes" |
| S13 | "proms" |
| S12 | (MH "Outcomes (Health Care)") OR (MH "Outcome Assessment") |
| S11 | (MH "Quality of Life") |
| S10 | S1 or S8 |
| S9 | S1 or S8 |
| S8 | S6 and S7 |
| S7 | S4 or S5 |
| S6 | S2 or S3 |
| S5 | AB allergy or allergic or hypersensitive or hypersensitivity or sensitive or sensitivity or intolerant or intolerance or reaction |
| S4 | TI allergy or allergic or hypersensitive or hypersensitivity or sensitive or sensitivity or intolerant or intolerance or reaction |
| S3 | AB food or nutrient |
| S2 | TI food or nutrient |
| S1 | (MM "Food Hypersensitivity") |

*Database: ISI Web of Science: Science Citation Index, Conference Proceedings Citation*

Search strategy:

Topic=((food or nutrient) AND (allergy or allergic or hypersensitive or hypersensitivity or sensitive or sensitivity or intolerant or intolerance or reaction)) AND Topic=((PROMs or PROM or patient reported outcome measure* or questionnaire* or HRQL or quality of life or health related quality of life or patient satisfaction or consumer satisfaction or patient preference or patient participation or "patient acceptance of healthcare" or patient outcome or patient based outcome or functional status or health status or subjective health status or health status indicator or health status assessment) AND (methodol* or psychometric* or validity or reliability or responsiveness or effect size or sensitivity to change or reproducibility or acceptability or utility measure*))

Timespan=All Years. Databases=CPCI-S.

Lemmatization=On
